# Selection of depression measures for use among Vietnamese populations in primary care settings: a scoping review

**DOI:** 10.1186/s13033-015-0024-8

**Published:** 2015-08-19

**Authors:** Jill Murphy, Elliot M. Goldner, Charles H. Goldsmith, Pham Thi Oanh, William Zhu, Kitty K. Corbett, Vu Cong Nguyen

**Affiliations:** Faculty of Health Sciences, Simon Fraser University, Blusson Hall, Room 11300, 8888 University Drive, Burnaby, BC V5A 1S6 Canada; Arthritis Research Centre of Canada, 5591 No. 3 Rd., Richmond, BC V6X 2C7 Canada; Institute of Population, Health and Development (PHAD), Alley No. 18, 132 Hoa Bang St., Cau Giay, Hanoi, Vietnam; Department of Psychological and Brain Sciences, Dartmouth College, 6207 Moore Hall, Hanover, NH 03755 USA; School of Public Health and Health Systems, University of Waterloo, Waterloo, ON N2L 3G1 Canada

**Keywords:** Vietnam, Depression, Measures, Primary care, Culture

## Abstract

Depression is an important and growing contributor to the burden of disease around the world and evidence suggests the experience of depression varies cross-culturally. Efforts to improve the integration of services for depression in primary care are increasing globally, meaning that culturally valid measures that are acceptable for use in primary care settings are needed. We conducted a scoping review of 27 studies that validated or used 10 measures of depression in Vietnamese populations. We reviewed the validity of the instruments as reported in the studies and qualitatively assessed cultural validity and acceptability for use in primary care. We found much variation in the methods used to validate the measures, with an emphasis on criterion validity and reliability. Enhanced evaluation of content and construct validity is needed to ensure validity within diverse cultural contexts such as Vietnam. For effective use in primary care, measures must be further evaluated for their brevity and ease of use. To identify appropriate measures for use in primary care in diverse populations, assessment must balance standard validity testing with enhanced testing for appropriateness in terms of culture, language, and gender and for acceptability for use in primary care.

## Background

While depression has been cited as a prominent and growing contributor to the global burden of disease [1], the construct and experience of depression may differ cross-culturally, with extensive variation in the expression of emotions, prominence of somatic symptoms and experience of and coping with suffering [2]. Despite this variation, evidence suggests that “depressive states can be studied as a feature of local forms of suffering” across the globe [2] and that symptoms associated with depression are present in all cultures [3]. The universality of depressive conditions combined with cross-cultural variation in the experience of depression mean that their identification and treatment are not culturally neutral. Strategies and tools that are culturally valid are essential.

The integration of mental health services into primary care is a recommended approach to address a large and growing gap in treatment [4, 5]. In the time-and resource-constrained context of primary care, identifying appropriate measures is needed to improve the use of limited resources and access to care [6].

Vietnam is a lower middle-income country with a diverse population of approximately 90 million people [7] and 54 distinct ethnic groups [8]. Over 4 million Vietnamese also live abroad [9]. Mental health services in Vietnam have been largely concentrated in tertiary psychiatric facilities and have focused on schizophrenia and epilepsy [10–12]. The availability of trained mental health practitioners in Vietnam is very low, with approximately 1 per 100,000 persons [13]. Primary health care service delivery is provided by 10,750 commune health stations that operate on a catchment system and staff approximately 47,000 primary care providers throughout the country [13]. The nature and scope of services and capacity in the primary care sector varies throughout the country. The government of Vietnam launched the National Community Mental Health Care (CMHC) project in 2001, with the goal of integrating mental health services into primary care. In practice, the CMHC emphasizes the provision of medications for patients with mental disorders although delivery and resources vary throughout the country [13]. Although depressive disorders are prevalent in Vietnam [11, 14, 15] evidence about their treatment is limited, suggesting that little has been done to build treatment capacity, especially at the primary care level. The government has recently shown interest in improving and expanding community-based services for depression [16].

This scoping review examines measures of depression that have been used in Vietnam or among the Vietnamese diaspora. The review assesses the validity of the measures as reported in the literature, taking into account the whether their cultural validity has been tested, the context in which they were validated or used, and their appropriateness for use in primary care with the objectives of: (1) making recommendations regarding the use of depression measures in primary care with Vietnamese populations and cross-culturally; (2) identifying gaps in the existing evidence.

## Review

Scoping reviews are appropriate to map “the relevant literature in the field of interest”, allowing for findings to be summarized and gaps identified [17]. Scoping reviews are rigorous, with methods that allow for replication, but findings are not synthesized or aggregated to the extent customary in systematic reviews. They allow for the inclusion of diverse study designs and involve an iterative search process where search terms may evolve during the review [17]. This method allows for identifying a broad range of studies.

We searched PubMed, PsychInfo and IDRC databases on June 12th and June 16th 2014. Search terms included: depression, mental disorder and mental health; scales, measures and screening; Vietnam and Vietnamese; we excluded ‘veterans’ given the many studies on American veterans [18]. Studies were included that validated or used measures deemed appropriate for use in general populations. The review included studies that explicitly validated instruments and those that used instruments in research, as several studies did not validate the instruments but nonetheless produced findings relevant to this review. Given the limited volume of research in this area, the search was not limited by date of publication.

**Fig. 1:**
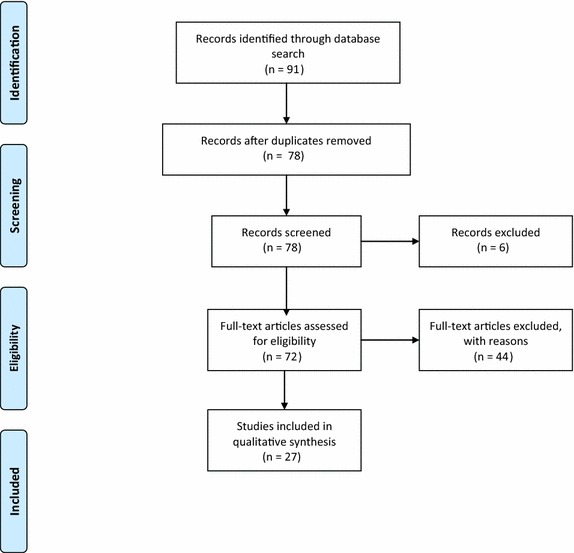
PRISMA diagram: search and selection process using Pubmed, PsychInfo and IDRC databases

Figure 1 shows the search and selection processes undertaken in this review. The database search identified 91 articles, which was reduced to 78 after removing duplicates. Initial screening eliminated 6 additional titles. Seventy-two full-text articles were reviewed for eligibility, with 44 then excluded. Studies were excluded because they: used data from the Vietnam Era Twin Study and were not related to Vietnamese populations; focused on neurological disorders or genetic markers; focused on specific rather than general populations or measures (e.g. scales to measure post-partum depression); or were not related to depression. Twenty-eight articles were first included in the review, with 11 measures initially identified. One measure and its source paper were excluded post hoc, since it compared mental health status in immigrants in Europe without discussing considerations specific to the measure or its use in Vietnamese populations [19]. The final set was 27 papers, with 10 measures identified.

Validation of psychometric measures is a broad methodological domain, with validity referring to “how well one can legitimately trust the results of a test as interpreted for a specific purpose” [20] or as “the ability of the instrument to measure the attributes of the construct under study” [21]. Validity typically addresses construct, content and criterion validity, and measures of reliability [20, 21]. Considerations such as semantic equivalence, which examines linguistic and idiomatic equivalence [22, 23], may also be included. Construct validity is the “degree to which an instrument measures the construct it is intended to measure” [21]. Content validity refers to whether the items in a measure reflect the full range of characteristics of the construct being examined. Criterion validity examines how a measure performs in relationship to another instrument or test. Finally, reliability examines the consistency with which a measure accurately identifies the construct of interest. Reliability is necessary but not sufficient for validity [20, 21]. Cook and Beckman [20] recommend that conclusions about an instrument’s validity should be based on several data types. The studies we reviewed employed various methods, some more comprehensive than others, to draw conclusions about validity.

Socio-cultural context is an essential consideration when assessing and understanding depression. This review includes measures that were developed for use in Western countries and subsequently applied with Vietnamese populations. We consider both the studies’ statistical results and qualitative observations about the appropriateness and cross-cultural validity of the measures in the context of the populations studied. We also consider factors influencing the appropriateness of measures for use in primary care. While the included studies did not all take place in primary care settings, characteristics of the measures, including the instrument’s length and accessibility, were identified as relevant to the measures’ appropriateness for use in such settings. Several measures assess common mental disorders (CMD) including depression, anxiety and somatic symptoms using sub-scales within one instrument. This analysis emphasizes the measurement of depression symptoms, but as symptoms of CMD often co-occur, attention is also given to the role that other symptoms might play in the Vietnamese experience of depression. Several studies validate or use more than one measure of depression. A list of the measures and their characteristics is found in Table 1.

**Table 1:** Overview of depression measures

Measure	Original author/citation	Context of initial development and use	Composition	Time to administer
Beck depression inventory (BDI)	Beck et al. [53]. Updated by Beck et al. [54]	Derived from clinical observations about the attitudes and symptoms displayed frequently by depressed psychiatric patients. Used widely in English-speaking populations	Self-administered or administered by trained interviewers. 21 symptoms of depression are rated from 0–3 in terms of intensity. Suggested cut-offs: minimal depression <10; mild to moderate depression 10–18; moderate to severe depression 19–29; severe depression 30–63	5–10 min
Centre for Epidemiologic Studies-Depression Scale (CES-D)	Radloff et al. [55]; revised by Eaton et al. [56]	Designed for epidemiological studies of depressive symptoms in the general population	Self-administered. Asks patients to identify current (this week) symptoms. 20 items, rated from 0 to 4 to indicate frequency of symptoms. Scored by summing ratings. Suggested cut-off: >16	10–20 min
Composite International Diagnostic Interview (CIDI)	WHO (1990). Updated by World Mental Health Survey [48]	Developed for use in low-cost, low-infrastructure settings	Self or interviewer administered yes/no items with 30 day recall period. 20 items. Suggested cut-off: 7/8. Algorithms for scoring provided only to trained administrators	5 min
Depression and Anxiety Stress Scale (DASS)	Lovibond and Lovibond [57]	Used to screen for depression, anxiety and stress in a community setting	42 items or 21 item short form. Three 7 question sub-scales for depression, anxiety and stress. 4 possible responses varying from 0 (did not apply to me at all) to 3 (applied to me very much or most of the time). For depression sub-scale cutoffs = mild: 5–6, moderate: 7–10, severe: 11–13, extremely severe: >14	5–10 min
Four Measures of Mental Health (FMoMH)	Beiser and Fleming [58]	Developed in Canada for use with South Asian refugee populations	50 items, 17 for depression, 16 for somatization, 13 for panic, and 4 for well-being. Study suggests cut-offs should vary by age and population	Not available
General Health Questionnaire (GHQ)	Goldberg [59]	Developed for use in primary care and non-clinical settings	Several versions: 60, 30, 28 and 12 items. GHQ-28 most frequently used. Four subscales: somatic symptoms, anxiety and insomnia, social dysfunction, and severe depression. Scales not independent of one another. Items are scored from 0 to 3 using alternative binary scoring, with a score of >4 considered indicative of psychological distress for the GHQ-28	5 min
Hopkins Symptom Checklist (HSCL)/Indochinese-HSCL	Parloff, Kelman, and Frank [60] revised 25-item version for use in primary care: Hesbacher et al. [61]. Indochinese version: Mollica et al. [62]	Originally developed as screening instrument for use in primary care and non-clinical settings. Was developed for use with Vietnamese refugees in the US	25 questions: 10 on anxiety subscale and 15 on depression subscale. Separate scores for anxiety, depression and total. There are 4 response categories, rated from 1 to 4. Maximum scores of 4 with 1.75 in clinical range	Not available
Phan Vietnamese Psychiatric Scale (PVPS)	Phan, Steel, and Silove [40]	Developed in Australia for use in Vietnamese diaspora	26 item depression subscale, a 13 item anxiety subscale, and a 14 item somatization subscale, with a total of 53 items. The depression scale includes two subscales: a 15 item affective subscale and an 11 item psycho-vegetative subscale. Items are scored from 1 to 3 based on frequency of occurrence. Cut-off for depression sub-scale = 1.85	Not available
SRQ-20	WHO [63]	Developed by WHO for use in low and middle income countries. Has been translated into many languages	Self or interviewer administered with 20 yes or no items with 30 day recall period. Yes responses are scored 1 and no responses are scored 0, with a maximum score of 20. Optimal cut-off considered to be 7/8	5–10 min
Vietnamese Depression Scale (VDS)	Kinzie [51]	Developed for use with Vietnamese refugees in the US	15 items with maximum score of 34. 6 items are culturally specific for Vietnamese population. Uses 3- and 4-point Likert scales. Optimal cut-off = >13	Not available

## Results

### Population

The studies reviewed measures used in Vietnam and among populations of Vietnamese living abroad. In several cases, different studies present data from the same study sample, while some present data from more than one study site or sample. Of the studies undertaken in Vietnam, seven present data from the north [24–30], five from the central provinces [6, 14, 24, 27, 31], and eight from the south [6, 15, 24, 27, 31–35]. In addition to studies conducted among general adult populations, two studies focused on older adults [35, 36], one on youth [14], five on mothers [24, 25, 27, 30, 37], one on men [28] and one on ‘Amerasians’, who are identified as a vulnerable population due to discrimination [38]. Of the studies taking place outside of Vietnam, one was in Taiwan among Vietnamese women [39], two were in Australia among adult immigrants and refugees [34, 40], and eight were in the United States (US), mostly with refugees or immigrants [38, 41–47]. One study identified the study population as Vietnamese Americans [47].

### Validation

The detailed results of the review are presented in Table 2. Of the ten measures found, eight were explicitly validated, including the: CES-D, CIDI, DASS, GHQ, iHSCL, PVPS, SRQ-20 and the VDS. Four of the measures were validated by one study each, while the iHSCL was validated by three studies (all in the US), the PVPS was validated by two (one in Australia and one in south Vietnam), the SRQ-20 was validated by three studies (in north, central and south Vietnam), and the VDS was validated by four (three in the US, one in south Vietnam). Of the validated measures, only five were validated for content validity (CES-D, CIDI, iHSCL, PVPS, SRQ-20), while seven were validated for construct validity (CIDI, DASS, GHQ, iHSCL, PVPS, SRQ-20, and VDS). Six measures were assessed for criterion validity (CIDI, DASS, iHSCL, PVPS, SRQ-20, and VDS) and four were tested for reliability (CES-D, DASS, PVPS, SRQ-20). The results of content, construct and criterion validity and reliability testing as assessed by each study are described in Table 2.

**Table 2:** Reported results on validity and reliability (unless otherwise reported, all values are for depression subscales)

Measure	Citation	Validated	Population	Sample size	Content validity	Construct validity	Criterion validity	Reliability
Beck Depression Inventory (BDI)								
	Lin and Hung [39]	No	VN immigrant women living in Taiwan	n = 143	Translated and back translated in VN	–	–	α = 0.80
Centre for Epidemiologic Studies-Depression Scale (CES-D)								
	Tran et al. [47]	Yes	VN immigrants in the United States	Boston community sample (n = 324); Nationwide sample (n = 452)	One item (“I felt that I was just as good as other people”) excluded for poor conceptual equivalence	–	–	Community sample α = 0.90; Nationwide sample α = 0.91
	Leggett et al. [64]	No	VN adults 55 years and older in Da Nang and surrounding areas	n = 600	Translated and back translated	–	–	α = 0.85
	Nguyen et al. [14]	No	Secondary students in Can Tho, Vietnam	n = 1161	–	–	–	–
	Gellis et al. [42]	No	VN immigrants in the US receiving psychiatric services	n = 79	–	–	–	α = 0.85 and α = 0.82 at two time points
	To et al. [35]	No	Older adults receiving cataract surgery in HCMC	n = 413 patients with 40 % loss to follow up	Translated and back translated	–	–	–
Composite International Diagnostic Interview (CIDI)								
	Rees et al. [33]	No	Random sample in one rural and one urban district in the MKD region	n = 3039	–	CIDI underreported depression prevalence (1.6 %) vs. the PVPS (7.4 %)	–	–
	Steel et al. (2009) [34]	Yes	VN population living in Vietnam and one living in Australia compared with an Australian-born population	n = 3039 in the MKD region, n = 1161 VN people living in Australia	Translated and back translated	MKD sample: CIDI and PVPS combined prevalence of 8.8 %. CIDI identified 42 unique cases, the PVPS 208, and both identified 16 cases. AUS sample: combined prevalence 11.7 %, CIDI indentified 38 cases, the PVPS 58 cases and both 40 cases	MKD Sample-depression subscale: AUC = 0.65 [95 % CI 0.56–0.73]. AUS sample-depression subscale: AUC = 0.73, (95 % CI 0.64–0.81)	–
	Liddell et al. [50]	No	A VN sample from the MKD, a VN immigrant population in Australia and an Australian-born sample	n = 3039 in the MKD, n = 1161 VN people living in Australia	CIDI translated and back translated	–	–	–
Depression and Anxiety Stress Scale (DASS)								
	Tran et al. [29]	Yes	Mothers in a rural northern Vietnam	n = 221	–	One factor (emotional state) significant, eigenvalue = 1.86	For cut-off of >10: Se: 80.8 %, Sp: 77.4 %; AUC 80.4 %	α = 0.72
	Nguyen et al. [14]	No	Students in Hue, Vietnam	n = 623	Translated and back translated	–	–	α = 0.81
	Fisher [25]	No	Pregnant women in Ha Nam, Vietnam	n = 6	–	–	–	–
Four Measures of Mental Health (FMoMH)								
	Phan, Steel and Silove [40]	No	Patients in Australia attending public psychiatric services and patients of general primary healthcare services	n = 86 psychiatric patients, n = 99 primary care patients	In cultural sensitivity questionnaire, participants responded: Words easy to understand = 30 %; Idioms that are familiar = 33 %; Individual questions constructed in meaningful way = 15 %; Symptoms that are similar to your or people you know = 15 %; Helpful in assisting a doctor identify mental illness: 22 %	MT-MM assessment showed a reliability of 0.94 for the depression subscale and showed that the 3 depression measures used in the study (PVPS, HSCL, FMoMH had the highest level of convergent validity	–	–
General Health Questionnaire (GHQ)								
	McKelvey, Webb, and Strobel [38]	Yes	“Amerasians” in Vietnam before their emigration to the US	n = 42 assessed for DSM-III depression, n = 5 cases	–	GHQ identified 2 of 5 DSM cases and 2 of 35 subjects without a DSM diagnosis as being in clinical range	.	–
Indochinese Hopkins Symptom Checklist (iHSCL)								
	Hinton et al. [43]	Yes	Newly-arrived adult VN refugees undergoing health screening in US	n = 206	Excluded item on loss of sexual interest, as deemed culturally inappropriate	–	Se = 86 %; Sp = 93 %; PPV = 48 %; AUC = 0.91 (SE = 0.06)	–
	Smith Fawzi et al. [46]	Yes	VN former POWs in US	n = 62	–	–	Using cut-off of 1.75: Se = 0.87; Sp = 0.7; AUC = 0.8916 (SD = 0.0448)	–
	McKelvey, Webb and Strobel [38]	Yes	“Amerasians” in Vietnam before emigration to the US	n = 42 assessed for DSM-III depression, n = 5 cases	–	The HSCL-25 identified 4 out 5 of the DSM-III diagnosed cases		–
	Phan, Steel and Silove [40]	No	Patients in Australia attending public psychiatric services and patients of general primary healthcare services	n = 86 psychiatric patients, n = 99 primary care patients	In cultural sensitivity questionnaire, participants responded: words easy to understand = 30 %; Idioms that are familiar = 34 %; Individual questions constructed in meaningful way = 18 %; Symptoms that are similar to your or people you know = 19 %; Helpful in assisting a doctor identify mental illness: 27 %	MT-MM assessment showed a reliability of 0.94 for the depression subscale and showed that the 3 depression measures used in the study (PVPS, HSCL, FMoMH had the highest level of convergent validity		
	McKelvey and Webb [38]	No	VN migrants to the US, pre and post migration	n = 161	–	–	–	–
Phan Vietnamese Psychiatric Scale (PVPS)								
	Phan, Steel and Silove [40]	Yes	Sample 1 recruited from mental health service and local primary care services and sample 2 recruited from two psycho-education classes in Australia	Sample 1: n = 185 and Sample 2: n = 64	Extensive review of traditional literature and an ethnographic survey. In cultural sensitivity questionnaire, participants responded: Words easy to understand = 43 %; Idioms that are familiar = 57 %; Individual questions constructed in meaningful way = 32 %; Symptoms that are similar to your or people you know = 32 %; Helpful in assisting a doctor identify mental illness: 42 %	CFA performed on responses from Study 1 and Study 2 showed four-factor model most appropriate (Chi square results decreased from 3858 for 1 factor model to 214 for 4 factor model in 1^st^ administration and from 3862 to 66 in 2nd administration). MT-MM showed reliability of 0.95 for the depression subscale and showed that the 3 depression measures used in the study (PVPS, HSCL, FMoMH) had the highest level of convergent validity compared with other sub-scales	Compared to psychiatrist diagnosis: Se = 83; Sp = 80; Overall agreement = 81; *K* = 0.62; PVPS cut-off = 1.95. Compared with DIS interview: Se = 84; Sp = 78; Overall agreement = 81; PVPS cut-off = 1.95. Compared with naturalist assessment: Se = 84; Sp = 87; Overall agreement = 85; PVPS cut-off = 1.85	Test–retest correlations for depression scale = 0.89; α for depression subscale in psychiatric sample: 0.93 at baseline and 0.94 at follow-up; in primary care sample: 0.95 at baseline and 0.95 at follow-up
	Steel et al. [34]	Yes	VN population living in Vietnam and one living in Australia, compared with an Australian-born population.	n = 3039 in the MKD region, n = 1161 VN people living AUS	–	MKD sample: CIDI and PVPS combined prevalence of 8.8 %. CIDI identified 42 unique cases, the PVPS 208, and both identified 16 cases. AUS sample: combined prevalence 11.7 % with the CIDI indentifying 38 cases, the PVPS 58 cases and both 40 cases	MKD sample- depression subscale: AUC = 0.65 [95 % CI 0.56–0.73]. AUS sample = depression subscale: AUC = 0.73, [95 % CI 0.64–0.81]	–
Self Reporting Questionnaire-20 (SRQ-20)								
	Tuan, Harpham and Huong [30]	Yes	Portion of random sample of female participants from child poverty study in Hung Yen, Vietnam.	n = 32 cases and n = 34 control	Translated and back translated		Compared with psychiatrist diagnosis (based on average of 3 interviews): Se = 73 %; Sp = 82 %; PCC = 79 %; AUC = 0.84 (95 % CI 0.75–0.94)	Inter-rater reliability at cut-off 7/8: *K* = 0.79 (z = 11.13, p < 0.001).
	Richardson et al. [31]	No	Adults in Da Nang and Khanh Hoa, Vietnam	n = 4981	–	–	–	–
	Son et al. [28]	No	Male MMORPG players in Hanoi, Vietnam	n = 344 players and n = 344 non-players	–	–	–	–
	Harpham et al. [27]	No	Mothers in 20 community sites in Vietnam	n = 1570	–	–	–	–
	Giang et al. [26]	Yes	Adult patients at district hospital and a sample from general community in rural northern Vietnam.	District hospital: n = 52. General community: n = 485	Translated and back translated. Researchers and health workers modified as needed. Piloted with patients, staff and community members at NIMH, district hospital and Bavi district. Report high face validity, but indicate that certain questions might lead to false “no” responses based on gender of respondent	–	A psychiatrist’s diagnosis using the CIDI used for comparison. Community sample: Optimal cut-off = 6/7; Se = 85 %; Sp = 61 %; Misclassification rate = 29 %; AUC = 0.86 [95 % CI 0.81–0.93]. Hospital sample: Optimal cut-off = 5/6; Se = 85 %; Sp = 46 %; Misclassification rate = 44 %; AUC = 0.74 [95 % CI 0.59- 0.89]	–
	De Silva et al. [24]	No	Primary caregivers of children in 20 semi-purposefully selected clusters in each of four countries (including Vietnam)	100 households in each cluster	–	–	–	–
	Stratton et al. [6]	Yes	VN adults in Da Nang and Khanh Hoa	n = 4980	–	1 factor EFA: CFI = 0.924; TLI = 0.915; RMSEA = 0.065; Bi-Factor Model: CFI = 0.977, TLI = 0.971, RMSEA = 0.030	–	α = 0.87
Vietnamese Depression Scale (VDS)								
	Hinton et al. [43]	Yes	Newly-arrived adult VN refugees undergoing routine, mandatory health screening in US	n = 206			Using cut-off of 12: Sp: 98 %; Se: 64 %; PPV: 75 %; NPV: 97 % (based on prevalence of major depression of 7 %). Cut-off of 11: Se = 79 %. AUC = 0.93 (SE = 0.05)	
	Dinh et al. [41]	Yes	Adult VN refugees in Houston, US	n = 180	–	PCA showed three factors with eigenvalues of >1, which were supported by the analysis of a Scree plot. For the three extracted factors: factor 1 (depressed affect): 40.8 % of variance, α = 0.92; factor 2 (somatic) 14.2 % of variance, α = 0.80; factor 3 (cultural specific) 10.1 % of variance, α = 0.81		–
	Nguyen et al. [15]	Yes	People who had already been diagnosed by a psychiatrist and patients presenting at a primary care clinic in HCMC, Vietnam	Previously-diagnosed patients: n = 115; Screened primary care patients: n = 177	Added item on sleep disturbance. Six items chosen more frequently by depressed subjects: feeling downhearted/low-spirited, low-spirited/bored, bothered and sad/bothered			
	McKelvey, Webb and Strobel [45]	Yes	“Amerasians” in Vietnam before emigration to the US	n = 42 assessed for DSM-III depression, n = 5 cases	–	–	VDS did not identify any DSM-III cases or other subjects as being in clinical range	–
	McKelvey and Webb [38]	No	VN migrants to the US, before and after migration	n = 161	–	–	–	–

### Characteristics of measures

While most studies did not assess the acceptability of the measures for use in primary care, the characteristics of the measures, including their brevity and accessibility, can help to inform decisions about their use in such settings. Of the validated measures, four were explicitly developed for use in non-clinical or community settings (DASS, GHQ, iHSCL and SRQ-20). The CES-D was developed for use in epidemiological studies, while the CIDI requires certification by the WHO-WMH, but training is unavailable in Vietnamese [48]. The PVPS is 53 items long, making it lengthy for use in time-sensitive settings. In the US contexts in which it was used, the iHSCL is described as easy to use, with clear language, and acceptable to target populations [43, 46] Based on its use in Vietnam, the brevity of the SRQ-20, the ease of administration and training, and cost-effectiveness are described as benefits of the measure [6, 31].

## Discussion

While there are numerous methods of assessing validity, definitions of “validation” and its associated methodology vary [20]. For the purposes of this review, we have taken into account the following criteria when assessing the extent to which validity of measures for use in primary care among Vietnamese populations has been tested: the extent to which a measure has been validated, including testing for construct and content validity in addition to criterion validity and reliability; the populations in which measures were tested, including the diversity of the populations and whether the measures were tested in Vietnam or among the diaspora; the length of the measures and ease of use.

While the review identified 11 studies that explicitly validated at least one measure of depression, the methods used and criteria on which conclusions were drawn were variable. While some studies reported minimally on content validity and reliability [47], others applied extensive testing [40]. The potential variation in cultural meaning and experience of depression in Vietnamese populations is an important issue that requires further study [40, 41, 49]. Consequently, testing only criterion validity and reliability does not assess whether depression as defined by the measure appropriately captures the culture-specific construct of depression. In studies that validate Western-developed measures for use in Vietnamese populations, assessing content and construct validity are critical aspects in determining if a measure is in fact valid. Many studies in this review examined the validity of measures in terms of their ability to determine caseness, but did not test the construct validity of the measure. Stratton et al.’s [6] psychometric evaluation of the Self Reporting Questionnaire-20 (SRQ-20) was a response to this gap in the literature. In addition, when criterion validity was assessed it was often based on comparison with international psychiatric diagnostic assessments [30, 40, 45] that have not been validated for use in Vietnam. The absence of somatic symptoms in these measures was associated with underreporting of depression in Vietnamese subjects [33, 50]. Assessing validity based on comparisons with these measures may be problematic.

The review included two measures that were developed for use among Vietnamese populations [40, 51] and one that was adapted for use among South Asian populations [41, 50], raising the question of whether indigenously derived measures are superior to adapted Western measures. The findings of this review suggest that a balance of culture-specific and universal constructs is essential for identifying depression in Vietnamese populations [41, 50]. Whether this can be accomplished sufficiently by modifying Western measures is debatable. In their work on depression in Zimbabwe, Patel et al. [52] found that Western derived measures could be used cross-culturally with sufficient attention to the translation of culture-specific concepts. All instruments would benefit from extensive evaluation of content and construct validity prior to being used in new populations.

This review includes studies that took place in Vietnam, or which took place among the Vietnamese diaspora. There are sizeable Vietnamese populations living in many countries, whose experience as migrants distinguishes them from Vietnamese living in Vietnam. The studies included in this review focused almost exclusively on immigrant or refugee populations and did not explicitly validate the measures for use among populations of Vietnamese descent living in these countries. While Vietnamese diaspora populations would certainly benefit from culturally appropriate measures of depression in primary care, consideration of the diversity of experience among these populations should be made when selecting appropriate measures. Vietnam itself is a diverse country with linguistic and cultural differences between regions, among rural and urban populations and among the diverse minority ethnic populations. For this reason, the validation of measures within specific populations should be considered before use. Many measures were only validated in one region of Vietnam or among one population sub-group. This should be considered when selecting measures for use in the country. Notably, no studies validated measures among minority population groups. This is a significant gap and additional research to validate measures for use in these populations is required.

Special considerations are also required for the use of depression measures in primary care environments, which often involve time and resource constraints and rely on non-specialist health staff. Measures must be brief, easy to use and to interpret, cost-effective and accessible. The PVPS, while extensively validated and based on a culturally relevant construct of CMD, is 53-items long with a 26-item depression subscale, making it lengthy for use in primary care environments.

Drawing definitive conclusions regarding the most appropriate measures for use among Vietnamese populations is difficult. The iHSCL may be most appropriate for use among immigrant or refugee populations. It includes 25 items, with a 15-item depression subscale, and has been found to be easy to use, with clear language. While it was found acceptable by refugees living in the US it has been only minimally tested in Vietnam and should be further validated for use in the country and for use among more established Vietnamese communities. The SRQ-20, which was validated and used in several regions of Vietnam, was found to be brief, easy for training and use and cost-effective [6], and thus appears most suitable for use in primary care settings in Vietnam. There are, however, concerns about differences between men and women when responding to the measure [6, 26] and it has not been validated among minority populations. Further research about differences in validity between urban and rural populations is also recommended.

### Limitations

This review includes only studies published in English. While we intended to include Vietnamese journals in this review, we found that as these journals are not indexed it was not feasible to include them. Further research in this area would benefit from including research published in Vietnamese journals. It was not possible to conduct a quantitative comparison across measures. The results therefore describe statistical results as reported in the studies and qualitative assessments.

## Conclusions

Assessing the validity of depression measures, their cultural acceptability and their appropriateness for use in primary care requires a balance of standard validity testing, assessment within the specific sociocultural context, and criteria such as brevity and cost-effectiveness. Vietnam is not only ethnically diverse but also has linguistic and cultural diversity by region that mean that, even when found to be valid in one population or region, a measure might require additional testing for use elsewhere. The increasing influence of Western culture, particularly in urban areas, may also lead to variation among the validity of measures between rural and urban populations. As demonstrated through testing of the SRQ-20, differences in gender in Vietnam may also mean that measures that can be used universally in Western populations will require gender-specific assessment in Vietnam.

The variability in methods used to validate instruments and populations in which the measures were tested makes definitive conclusions about the most effective measure difficult. Studies or services wishing to implement depression screening in primary care have an extensive body of literature on which to draw to make decisions about the most appropriate instrument for their purposes. Specific consideration, however, to population, region and gender specific factors should be given when using these measures in practice. Pilot testing of measures validated in one region or population to be used in another is recommended in order to ensure conceptual and linguistic equivalence of measures across the diverse Vietnamese population.

While a discussion of the validation of depression measures in other contexts was beyond the scope of this review, the findings have implications beyond Vietnamese populations. We found little consistency in the validation of depression measures, with limited attention to construct and content validity, both of which are important to determine cross-cultural validity. To guide further studies of validation of psychometric measures in cross-cultural contexts and in primary care, enhanced methodological guidelines for validation, which include testing of all types of validity and which draw on anthropological methods to avoid pitfalls of testing against non-validated Western psychiatric assessments, would benefit researchers conducting validation studies and health services wishing to employ the most appropriate measure.

## Abbreviations

### Depression measures

BDI: Beck Depression Inventory; CES-D: Centre for Epidemiologic Studies-Depression Scale; CIDI: Composite International Diagnostic Interview; DASS: Depression and Anxiety Stress Scale; FMoMH: Four Measures of Mental Health; GHQ: General Health Questionnaire; HSCL: Hopkins Symptom Checklist; iHSCL: Indochinese Hopkins Symptom Checklist; PVPS: Phan Vietnamese Psychiatric Scale; SRQ-20: Self-Reporting Questionnaire-20; VDS: Vietnamese Depression Scale

### Statistical tests

Se: sensitivity; Sp: specificity; AUC: area under the receiver operating curve; PPV: positive predictive value; NPV: negative predictive value; PCC: probability of correct classification; *K*: kappa coefficient; EFA: exploratory factor analysis; CFI: Comparative Fit Index; TLI: Tucker–Lewis Index; RMSEA: root mean square error of approximation; PCA: principal component analysis; MT-MM: multitrait-multimeasure

### Contextual acronyms

NIMH: National Institute of Mental Health; HCMC: Ho Chi Minh City; VN: Vietnamese; MMORPG: Mix multi-player online role playing game; MKD: Mekong Delta Region; IDRC: International Development Research Centre; DSM: Diagnostic and Statistical Manual; US: United States; WHO: World Health Organization; POW: Prisoner of War; DIS: Diagnostic Interview Schedule for DSM
